# Non-invasive methods for the assessment of biomarkers and their correlation with radiographic maturity indicators — a scoping review

**DOI:** 10.1186/s40510-021-00372-6

**Published:** 2021-09-06

**Authors:** Veena GV, Tulika Tripathi

**Affiliations:** Department of Orthodontics and Dentofacial Orthopaedics, Maulana Azad Institute of Dental Sciences, Bahadur Shah Zafar Marg, New Delhi, 110002 India

**Keywords:** Saliva, Gingival crevicular fluid, Urine, Biomarker, Puberty, Maturity

## Abstract

**Background:**

Detection of skeletal maturity is vital in orthodontic treatment timing and planning. Traditional methods include hand-wrist radiography and cervical vertebral maturation index (CVMI). Though the radiographic methods are well established and routinely used to assess skeletal maturation, they carry the drawback of subjective perception and low reproducibility. With evolving concepts, skeletal maturation has been assessed quantitatively through biomarkers obtained from saliva, gingival crevicular fluid (GCF), and urine. The scoping review aims to explore the various biomarkers assessed through non-invasive methods and their correlation with radiographic skeletal maturity.

**Methodology:**

The literature search was carried out on MEDLINE via Pubmed, Cochrane Library (Cochrane database of systematic reviews), Cochrane central register of controlled trials (CENTRAL), Google Scholar, Semantic Scholar, ScienceDirect, and Opengrey.eu for articles up to and including November 2020. Pertinent articles were selected based on inclusion and exclusion criteria. The results were tabulated based on the type of sample collected, the biomarker assessed, method of sample collection, and the radiographic method used.

**Results:**

The literature search resulted in 12 relevant articles. Among all the studies, 10 studies showed that the concentration of biomarkers increases during the pubertal growth peak. On the contrary, 2 articles showed no significant difference between the levels of biomarkers and pubertal growth peak.

**Conclusion:**

It can be concluded that the level of biomarkers increases during the pubertal growth spurt and can provide a quantitative way of assessing skeletal maturity.

## Introduction

Assessment of maturation with particular regard to the onset of pubertal growth provides critical information about the likelihood of growth changes occurring in the craniofacial structures. This has a bearing on timing the orthodontic treatment by utilizing the growth potential, especially when dealing with skeletal disharmonies. Numerous methods have been developed to assess the developmental status, among which skeletal maturity is most widely used. This could be assessed through several biological indicators like height [1–3], weight [4], chronological age [5, 6], skeletal maturation of hand-wrist [7–9], and changes in the morphology of cervical vertebrae [10–14].

Hand-wrist radiographic method, initially developed by Todd [15] and followed by various authors [16, 17], is a well-established method for assessing skeletal maturation. It is based on various morphological changes and ossification events occurring in the carpal bones. In spite of its accuracy, the major drawback resides in the requirement of an additional radiograph. Lateral cephalogram radiograph is a routine orthodontic investigation. Assessment of skeletal maturation through cervical vertebral maturation index (CVMI) was a technique described by Lamparski [12] in 1972 and later modified by Baccetti et al. [10].

CVMI, albeit widely used, suffers from low reproducibility, subjective perception of the practitioner [18, 19], issue in staging cephalometric radiographs with radiographic noise, complexity in the identification of landmarks, and borderline subjects [20]. Further, it has been established that the cervical column differs in numerous skeletal jaw relationships and pressure; morphology of facial components and body posture alter the height of vertebral bodies and thus have an effect on the reliability of CVMI.

Newer possibilities are provided with biochemical markers that are involved in bone growth and remodeling [21, 22]. An increase in their levels is correlated to the peak circumpubertal growth [22]. These biomarkers can be procured from various biological fluids such as blood, saliva, gingival crevicular fluid (GCF), and urine. They permit the possibility of quantification, overcoming the subjective errors associated with the radiographs.

Saliva, GCF, and urine have the advantage of avoiding invasive blood collection. It is additionally useful in clinically challenging situations such as obtaining samples from children or anxious patients where blood sampling might be a difficult act to perform. The primary goal of the present article was to identify and summarize the biomarkers assessed through non-invasive methods and their correlation with radiographic maturity indicators.

## Methodology

The literature search was carried out in MEDLINE via Pubmed, Cochrane Library (Cochrane database of systematic reviews), Cochrane central register of controlled trials (CENTRAL), Google Scholar, Semantic Scholar, ScienceDirect, and Opengrey.eu for articles up to and including November 2020. The keywords used were saliva, GCF, Gingival Crevicular fluid, urine, biomarker, marker, pubert*, matur*, cervical vertebra*, radiograph*, hand-wrist, skeletal age. The titles and abstracts of the articles found were read to match the inclusion criteria. The inclusion criteria were (a) human studies that used non-invasive methods for assessing biomarker, (b) studies assessing skeletal maturity/pubertal growth spurt, and (c) studies using cervical vertebral maturation index or hand-wrist methods as their radiographic indicator. The exclusion criteria were (a) studies that collected serum samples, (b) animal studies, (c) reviews, (d) studies conducted on subjects with systemic conditions, and (e) articles in languages other than English. The search resulted in 682 articles. Removal of duplicates and the articles pertinent to the inclusion and exclusion criteria were evaluated.

## Results

A total of 682 articles, following the database search, were reviewed. The redundant articles were removed. Twelve articles were selected for full-text review and summarized based on the following information: biomarker assessed, sample size and characteristics, sample collection protocol, processing of sample, biomarker detection method, and the radiographic method used (Table 1). All 12 studies were cross-sectional and included subjects of both the genders except two studies [28, 34] that only had female participants. The age group in the studies ranged from 6 to 24 years. The non-invasive methods used were saliva [23–30], GCF [31–33], and urine [34].

**Table 1:** Characteristics of samples, method of collection and processing, biomarker detection technique, radiographic method used, and conclusions

	Study	Type of study	Sample	Age group	Gender	Type of sample	Method of sample collection	Parameters assessed	Sample processing	Biomarker detection technique	Radiographic method used	Conclusion
**Saliva**												
IGF1	Nayak et al. [23]	Cross-sectional	45 subjects	7 to 23 years	24 females, 21 males	Unstimulated parotid saliva	Saliva was collected with a modified Lashley cup for a duration of 30 min.	a. Salivary flow rate (ml/min) b. IGF1 levels(ng/ml) c. IGF1 secretion rate (ng/min)	Centrifugation for 10 min at 1500×*g* at room temperature, and stored at – 20 °C until analysis.	Immunoradiometric assay (IRMA)	Lateral cephalogram—Quantitative Cervical Maturation System (QCVM) by Chen et al. (2008)	Results showed that the IGF1 levels in saliva and the rate of secretion were lower in QCVM I followed by an increase in QCVM II and a decrease in QCVM III and IV.
BALP	Wijaya et al. [24]	Cross-sectional	136 subjects	8 to 18 years	64 males, 72 females	Unstimulated whole saliva at 9 am	Passive drooling method, after distilled water mouth rinse	a. Total protein content b. BALP levels(pg/ml)	Samples were centrifuged and stored in the icebox until analysis.	Enzyme-linked immunosorbent assay (ELISA)	Lateral cephalogram—Cervical Vertebral Maturation Index by Baccetti et al. [10]	No significant differences were found between the groups.
	Hegde et al. [25]	Cross-sectional	90 subjects	6 to 19 years	Not specified	Unstimulated whole saliva	Passive drooling method	a. Salivary BALP levels(U/I)	Not specified.	Enzyme-linked immunosorbent assay (ELISA)	Hand-wrist radiographs—Hagg and Taranger	They concluded that the BALP levels in saliva increased in peak pubertal stage and showed a gradual increase from Subgroup S_0_ to Subgroup MP3.
ALP	Alhazmi et al. [26]	Cross-sectional	79 subjects	7 to 23 years	48 females, 31 males	Unstimulated whole saliva was collected between 9 am to 12 pm	Passive drooling method for a period of 5 min	a. Salivary ALP activity (mU/mg) b. Total protein concentration (mg/mL)	Samples were centrifuged and stored at – 80 °C until analysis.	Colorimetric assay	Lateral cephalograms—Cervical Vertebral Maturation Index by Baccetti et al. [10]	- Protein concentration was higher in CVMS III and V. - Higher ALP levels in saliva were found in CVMS I. The salivary activity of ALP was higher in males, suggesting their increased growth potential and longer growth spurt duration than in females.
	Tarvade et al. [27]	Cross-sectional	120 subjects	10 to 15 years	60 males, 60 females	Unstimulated whole saliva	Not specified	a. Salivary ALP levels (IU/L)	Samples were centrifuged and stored in an icebox before analysis.	Colorimetric assay	Middle phalanx third finger—MP3 staging by Hagg and Taranger	The levels of salivary ALP reached a peak in the G stage of MP3 followed by a decrease in the H stage. They also found that both boys and girls showed ALP levels correlating well with the G stage of MP3
	Irham et al. [28]	Cross-sectional	57 subjects	8 to 15 years	57 females	Unstimulated whole saliva was collected between 10 am to 12 pm	Not specified	a. Salivary ALP levels (IU/L)	Samples were centrifuged for 2 min for 10,000 rpm and stored at – 80 °C.	Colorimetric assay	Lateral cephalogram—Cervical Vertebral Maturation Index by Hassel and Farman	Increased activity of salivary ALP was seen in the pubertal phase. The difference between ALP levels of pre-pubertal and pubertal, and pubertal and post-pubertal was statistically significant.
VEGF	Sharmila et al. [29]	Cross-sectional	90 subjects	6 to 20 years	Not specified	Unstimulated whole saliva	The subjects were asked to expectorate saliva in a 15-ml Tarzon centrifuge tube after retaining it in the mouth for 5 min.	a. Salivary IGF1 levels (ng/ml) b. Salivary VEGF levels (pg/ml)	Centrifugation for 10 min at 1500×*g* at room temperature and stored at – 20 °C until analysis.	Enzyme-linked immunosorbent assay (ELISA)	Lateral cephalogram—Cervical Vertebral Maturation Index by Hassel and Farman	No statistical difference was found among the pre-pubertal, pubertal, and post-pubertal stages for VEGF. But IGF1 levels were found to increase in the pubertal growth phase.
DHEA	Sangeeth et al. [30]	Cross-sectional	66 subjects	9 to 18 years	33 males, 33 females	Unstimulated whole saliva was collected at 10 am	Passive drooling in a plastic vial	a. Salivary DHEA levels (pg/ml)	Centrifugation at 3000 rpm for 15 min. The samples were stored at – 4 °C immediately after collection (within 30 min) and transferred to – 20 °C within 4 h of collection.	DHEA Immuno Assay	Lateral cephalogram—Cervical Vertebral Maturation Index by Baccetti et al. [10]	They found a gradual increase in DHEA concentration in saliva from stage 1 to stage 6 with the highest levels found in stage 5 and 6.
**GCF**												
ALP	Perinetti et al. [31]	Cross-sectional	72 subjects	7.8–17.7 years	45 females, 27 males	GCF collected using #25 standardized sterile paper strips inserted 1 mm into the gingival crevice and left in situ for 60 s	Two sites on each maxillary and mandibular central incisor	a. GCF ALP activity (mU/sample)	Samples transferred to plastic vials and immediate storage at – 80 °C until analysis.	The total ALP activity was determined by monitoring the increase in spectrophotometric absorption, at 405 nm. The absorbance was converted into enzyme activity units (1 unit 5 1 mmol of p-nitrophenol released per minute at 37°C) and expressed as total activity in mU per sample.	Lateral cephalogram—Cervical Vertebral Maturation Index by Baccetti et al. [10]	A twofold increase in GCF ALP activity was found during the pubertal phase than during the pre-pubertal and post-pubertal phases.
	Perinetti et al. [32]	Cross-sectional	50 subjects	7.8–17.7 years	31 females, 19 males	GCF collected using #25 standardized sterile paper strips inserted 1 mm into the gingival crevice and left in situ for 60 s	Two sites on each maxillary and mandibular central incisor	a. Total protein content (μg/sample) b. Total GCF ALP activity (mU/sample) c. Normalized GCF ALP activity (mU/μg proteins)	Four samples were pooled and immediately stored at – 80 °C	The total ALP activity was determined by monitoring the increase in spectrophotometric absorption, at 405 nm. The absorbance was converted into enzyme activity units (1 unit 5 1 mmol of p-nitrophenol released per minute at 37°C) and expressed as total activity in mU per sample.	Lateral cephalogram—Cervical Vertebral Maturation Index by Baccetti et al. [10]	The total activity of GCF ALP was found to be significantly higher in the pubertal growth phase than in the pre-pubertal and post-pubertal growth phases in both the maxillary and mandibular sites.
VIT DBP	Wen et al. [33]	Cross-sectional	40 subjects	6.8–24 years	20 males, 20 females	GCF was collected between 8 am and 10 am. Paper strips were inserted and left in situ for 30 s.	From mesiolabial and distolabial sites of upper central incisors	a. Vitamin D binding protein (μg) b. Serotransferrin (μg)	50 μl phosphate-buffered solution was added into the Eppendorf tubes containing four paper points and shaken at 4 °C for 10 min and then centrifuged at 9000 rpm for 5 min.	Enzyme-linked immunosorbent assay (ELISA)	Cervical vertebral maturation method proposed by Franchi et al. [35]	GCF levels of vitamin DBP and Serotransferrin were found to be significantly greater in pubertal compared to the post-pubertal growth phase.
**Urine**												
IGF1	Sinha et al. [34]	Cross-sectional	72 subjects	8–20 years	72 females	Random morning midstream urine samples were collected		a. Urine IGF1 levels (ng/ml)	The urine samples were pipetted in Eppendorf tubes and ultracentrifuged and stored at – 80 °C	Enzyme-linked immunosorbent assay (ELISA)	Lateral cephalogram—Cervical Vertebral Maturation Index by Hassel and Farman	Increased urinary IGF1 levels were found in CVMI stage 4 corresponding to the mean age of 13.67 years.

### Saliva

Out of the 12 articles, 8 studies [23–29] employed saliva as their sample. The biomarkers assessed in the eight studies were insulin-like growth factor 1 (IGF1) [23, 29], alkaline phosphatase (ALP) [26, 27, 34], bone-specific alkaline phosphatase (B-ALP) [24, 25], vascular endothelial growth factor (VEGF) [29], and dehydroepiandrosterone (DHEA) [30].

Hegde et al. [25] and Tarvade et al. [27] used hand-wrist radiographic method for correlation, while the remaining six studies used CVMI as their radiographic indicator.

Nayak et al. [23] found IGF1 levels to increase during the period of accelerated velocity (QCVM II).

Salivary ALP levels were assessed in three studies. Irham et al. [28] found a significant increase in ALP levels during pubertal phase. The study by Tarvade et al. [27] that used middle phalanx of the third finger (MP3) staging to correlate found the ALP levels to increase during G stage of MP3. However, Alhazmi et al. [26] concluded that the ALP levels were higher only during the CVM stage 1.

The study by Wijaya et al. [24] which assessed the BALP levels did not find any significant difference across the various CVMI groups. On the contrary, Hegde et al. [25] concluded the BALP levels to increase during the peak pubertal phase (MP3-G stage).

VEGF and IGF assessed by Sharmila et al. [29] did not find any significant difference in VEGF levels across the various CVMI stages but found the IGF1 levels to increase during the pubertal growth phase.

A gradual increase in DHEA levels from CVMI stage 1 to stage 6 was found by Sangeeth et al. [30] with the highest levels during stages 5 and 6.

### GCF

Three studies [31–33] collected GCF. Perinetti et al. [31, 32] correlated the GCF ALP activity using CVMI. They found a twofold increase in GCF ALP levels during the pubertal phase. Wen et al. [33] found the levels of vitamin D binding protein (VIT DBP) and serotransferrin (Tf) to increase during pubertal growth phase (CVM 3 and 4) compared to the other proteins detected.

### Urine

Only one study collected urine [34]. The biomarker assessed was IGF1 using CVMI radiographic indicator. The study was conducted only in female subjects. They found an increase in IGF1 levels during CVMI stage 4.

## Discussion

The well-known intra-individual variations in the concentration of biomarkers throughout the various anabolic and catabolic processes preclude their use for diagnostic purposes. Saliva, GCF, and urine hold the benefit of being non-invasive methods.

### Saliva

Out of the twelve studies reviewed in this article, 8 studies collected saliva samples. It was noticed that every study adopted its own protocol of procedures for sample collection such as time of collection, refraining from eating and drinking, before or after tooth brushing, and oral hygiene. Most of the studies instructed the patients to refrain from eating and drinking 30 to 90 min before collection. In all the studies, there is a strong consensus in the collection of unstimulated saliva. In salivary diagnostics, though it is well documented that there is an increase in the salivary flow following stimulation [36], it contains only a diluted concentration of biomarkers that might be tough to detect [37]. Hence, unstimulated saliva is usually preferred. However, unstimulated saliva is affected by factors like the degree of hydration, position of the head during collection, drugs, and circadian rhythm. Stimulation of parotid salivary flow with 5% citric led to a decrease in the concentration of IGF1 considerably [38].

In terms of the site of sample collection, all studies collected the whole saliva except Nayak et al. [23] that collected parotid saliva. Literature suggests collecting whole saliva is an easier and feasible method [39].

Six out of 8 studies collected saliva by a passive drooling method. Nayak et al. [23] collected saliva with a modified Lashley cup over the parotid gland and Sharmila et al. [29] by saliva accumulation for 5 min and then expectorating it. In the spitting method, 14 times more bacteria were present in samples compared to the drooling method. This could affect the storage and analysis of proteins [40].

### Gingival crevicular fluid

The constituents of gingival crevicular fluid include host-derived enzymes, plasma and serum components, inflammatory cells, and immune cells. In healthy state, the protein concentration in GCF is found to be similar to the interstitial fluid [41]. All the three studies collected GCF by absorbent paper strip technique as this method is quick and easy and also because of the ability of the strips to quickly absorb the fluid [41]. In two studies, samples were taken from two sites of maxillary and mandibular central incisors, whereas in one study samples were taken from the mesiolabial and distolabial aspect of only maxillary central incisors.

### Urine

Though urine can be collected in large volumes repeatedly, limited literature is available on its use for detecting biomarkers related to skeletal maturity. Urine proteomics differ depending upon factors like age, gender, diet, hormonal status, and circadian rhythm [42]. Study conducted by Sinha et al. [34] collected random morning midstream urine sample only in female subjects.

### Circadian flow

The time of sample collection will vary the result greatly due to the effect of the circadian rhythm on the protein concentrations [43]. IGF1 demonstrates little diurnal variations with levels peaking slightly during morning hours [38]. The B-ALP is the only marker not influenced by circadian rhythm due to its molecular structure [44]. DHEA showed considerable diurnal variation with higher hormonal levels in the morning than later in the day. Among the 12 studies, 6 collected samples in the morning.

Among the 12 articles reviewed, 3 studies employed IGF1 as their biomarker out of which two studies were conducted in saliva and one in urine. The quantification of growth hormone (GH) becomes intricate due to its strong binding capacity to GH binding protein, providing unreliable information about the functionally active GH. The continuous diurnal variation also abuts to its difficulty in estimation. Longitudinal studies have enunciated the direct modulating effects of IGF1 on GH [45]. It has an influence on bone growth and formation, showing variations periodically during bone growth. Nayak et al. [23] measured the levels of IGF1 in saliva and correlated it with the quantitative cervical vertebral maturation index. They concluded that the levels increased during the period of accelerated velocity. Similarly, the results of Sharmila et al. [29] showed the levels of salivary IGF1 to reach a peak during the pubertal stage. This was in agreement with previous studies which correlated the serum IGF and found peak levels during pubertal growth [34, 46–52]. Few studies also found a disparity in IGF1 levels in males and females across the CVMI stages as IGF1 levels are influenced by sex [53]. Sinha et al. [34] conducted the study only on female subjects to avoid gender bias and noted the urinary IGF1 levels to increase during CVMI stage 4, corresponding to the mean age of 13.67 years. Since these studies have emphasized that the salivary and urine IGF1 levels follow a similar pattern of secretion rate to serum IGF, they can be used as an indicator of residual mandibular growth.

Alkaline phosphatase activity was correlated in 5 [26–28, 31, 32] out of 12 studies. Salivary ALP activity was measured in three studies. Alkaline phosphatase catalyzes the hydrolysis of phosphate ester groups and helps in hydroxyapatite crystallization. It is widely used as a biomarker for pathological states of liver and bone. Irham et al. [28] and Alhazmi et al. [26] compared it with CVMI staging while Travade et al. [27] used the Hagg and Taranger method (ossification of middle phalanx of the third finger-MP3) as their radiographic indicator. This method evaluates skeletal maturity based on the capping of epiphysis of the middle phalanx of the third finger. They are divided into five stages: MP3 F, MP3 FG, MP3 G, MP3 H, and MP3 I. MP3FG stage indicates acceleration towards pubertal peak; G stage indicates the peak pubertal phase followed by a deceleration H stage. Alhazmi et al. [26] noted the salivary ALP levels to be higher in CVMI 1 and males. They suggested that this could be due to the increased growth potential and long growth spurt duration in males than in females. They also found that the total protein concentration was higher in CVMI 3 and 5. Irham et al. [28] conducted the study only on female subjects and found the ALP levels to rise during the pubertal phase. The levels in the study by Travade et al. [27] showed a peak in the G stage of MP3.

Two studies [31, 32] determined the levels of ALP in GCF and correlated it with CVMI stages. Perinetti et al. [31] found a twofold increase in levels during the pubertal growth phase. The other study [32] found an increase in total enzyme activity (mU/sample) during C3 and C4 stages. No significant difference was found on correlating the normalized GCF ALP activity (mU/μg) to CVMI stages, thus eliminating it as an indicator for skeletal maturation.

Bone-specific ALP (B-ALP), an isozyme of ALP, is an important product of osteoblasts involved in the process of bone mineralization [44]. It is a more sensitive marker that becomes readily detectable even with minor fluctuations. BALP levels were assessed by Hegde et al. [25] and Wijaya et al. [24] in saliva. Hedge et al. [25] concluded that the levels increased in peak pubertal stage and showed a gradual increase from subgroup S_o_ to MP_3_. The results were consistent with the studies which correlated serum BALP levels with CVMI in males and females [49]. Serum BALP correlated with Tanner’s pubertal stages and chronologic age also revealed similar results [44, 54, 55]. However, the study by Wijaya et al. [24] revealed results on the contrary showing no significant differences between pre-pubertal and pubertal groups.

The vascular endothelial growth factor is a potent angiogenic and an essential growth factor of vascular endothelial cells [56]. Zelzer et al. first described the connection between VEGF and chondrocytes during skeletal development [57]. An in vitro study on human osteoblast cells suggested that VEGF promotes osteoblasts and alkaline phosphatase activity. The possible non-vascular activities of VEGF in bone ossification have led to a presumption that it might have a role in skeletal maturity.

Nevertheless, the study by Sharmila et al. [29] found no significant difference in salivary VEGF levels among the pre-pubertal, pubertal, and post-pubertal stages, and the levels were found to decrease with pubertal and post-pubertal stages. Further studies need to be carried out to substantiate it as a biomarker for assessing skeletal maturity.

Dehydroepiandrosterone (DHEA) is an intermediate during the synthesis of androgen and estrogen [58]. They improve bone mineral density. Studies have emphasized that the first peak in DHEA levels occur between 6 and 7 years of age, the second peak during puberty, and gradually increases till it reaches adult values [59–61].

Sangeeth et al. [30] found that the DHEA levels in saliva increased from stage 1 to stage 6. Similar results were obtained with the study conducted by Netherton et al. [62] and Matchock et al. [63] that correlated the levels with Tanner’s pubertal growth stages. Srinivasan et al. [64] correlated the serum levels of DHEA with hand-wrist methods and found an increase during the pubertal growth phase.

The study conducted by Wen et al. [33] detected a total of 537 proteins in GCF among which the levels of vitamin D binding protein and serotransferrin were found to be significantly higher during the pubertal growth phase. An increase in serum levels of transferrin in boys during puberty was reported by Antilla et al. [65]. No other studies in the literature have correlated vitamin DBP to skeletal maturity.

### Scope for future research

It is critical to harness the full growth potential of an individual to reduce the burden of surgical intervention at a later stage. Using saliva, GCF, and urine for assessing skeletal maturity is practical, non-invasive, and repeatable. Despite its advantages, the studies cited above assessing the same biomarker through same biologic fluid had deviations in the sample collection method, time of collection, processing, and storage temperature. All these could affect the reproducibility and sensitivity of non-invasive methods. Furthermore, longitudinal studies with standardization of protocol would be expedient to conclude any biomarker as a skeletal maturity indicator.

Studies indicate that growth factors like VEGF and IGF-1 play a major role in the proliferative activity of condylar cartilage in skeletal class III malocclusion. Translational research identifying the protein expression in different malocclusions will provide a rationale for diagnosis in orthodontics and dentofacial orthopedics.

Till date, one of the major challenges is the development of a robust, versatile, and sensitive clinical tool for chairside practice which would warrant the use of non-invasive methods for assessing skeletal maturity.

## Conclusion

Considerable literature on the diagnostic possibilities of saliva, GCF, urine, and their implications in orthodontics and dentofacial orthopedics is available. Unlike radiographs, assessing skeletal maturity through non-invasive methods can be repeated at much shorter intervals and the biomarkers can be quantified. There is adequate agreement among the available studies that the level of certain biomarkers peak during the pubertal growth phase (i.e., C3 and C4 of CVMI and G stage of MP3). However, significant drawbacks of saliva collection are variation in the method, processing, and storage temperature which may affect the biomarker levels. GCF is technique sensitive and contamination with blood and saliva can occur during collection. Also, different biomarkers require different assay. If effort is taken to mitigate all the abovementioned issues, the heterogeneity can be minimized, and clinical effectiveness can be greatly improved. The scope is vast but the standardization of procedures still remains to be further investigated.

## Data Availability

Not applicable

## Abbreviations

CVMI: Cervical Vertebral Maturation Index
GCF: Gingival crevicular fluid
IGF1: Insulin-like growth factor 1
ALP: Alkaline phosphatase
B-ALP: Bone-specific alkaline phosphatase
VEGF: Vascular endothelial growth factor
DHEA: Dehydroepiandrosterone
VIT DBP: Vitamin D binding protein
Tf: Serotransferrin
QCVM: Quantitative Cervical Vertebral Maturation Index
GH: Growth hormone
MP3: Middle phalanx of the third finger
ELISA: Enzyme-Linked Immunosorbent Assay
IRMA: Immunoradiometric assay
